# Quantification of blood and CSF volume to predict outcome after aneurysmal subarachnoid hemorrhage

**DOI:** 10.1007/s10143-024-03001-y

**Published:** 2024-10-08

**Authors:** James Booker, Ardalan Zolnourian, James Street, Mukul Arora, Anand S Pandit, Ahmed Toma, Chieh-Hsi Wu, Ian Galea, Diederik Bulters

**Affiliations:** 1 Department of Neurosurgery, Wessex Neurological Centre, Southampton General Hospital, Southampton, SO16 6YD UK; 2 Wellcome/EPSRC Centre for Interventional and Surgical Sciences, University College London, London, UK; 3 Victor Horsely Department of Neurosurgery, National Hospital for Neurology and Neurosurgery, London, UK; 4 Institute of Behavioural Neurosciences, University College London, London, UK; 5 Faculty of Medicine, University College London, London, UK; 6 School of Mathematical Sciences, University of Southampton, Southampton, UK; 7 Clinical Neurosciences, Clinical and Experimental Sciences, Faculty of Medicine, University of Southampton, Southampton, UK

**Keywords:** Subarachnoid hemorrhage, Aneurysm, Machine learning, Image segmentation

## Abstract

This study aimed to describe the relationship between blood and CSF volumes in different compartments on baseline CT after aSAH, assess if they independently predict long-term outcome, and explore their interaction with age. CT scans from patients participating in a prospective multicenter randomized controlled trial of patients with aSAH were segmented for blood and CSF volumes. The primary outcomes were the mRS, and the Subarachnoid Hemorrhage Outcome Tool (SAHOT) at day 28 and 180. Univariate regressions were conducted to identify significant predictors of poor outcomes, followed by principal component analysis to explore correlations between imaging variables and WFNS. A multivariate predictive model was then developed and optimized using stepwise regression. CT scans from 97 patients with a median delay from symptom onset of 271 min (131–547) were analyzed. Univariate analysis showed only WFNS, and total blood volume (TBV) were significant predictors of both short and long-term outcome with WFNS more predictive of mRS and TBV more predictive of SAHOT. Principal component analysis showed strong dependencies between the imaging predictors. Multivariate ordinal regression showed models with WFNS alone were most predictive of day 180 mRS and models with TBV alone were most predictive of SAHOT. TBV was the most significant measured imaging predictor of poor long-term outcome after aSAH. All these imaging predictors are correlated, however, and may have multiple complex interactions necessitating larger datasets to detect if they provide any additional predictive value for long-term outcome.

**Supplementary Information:**

The online version contains supplementary material available at 10.1007/s10143-024-03001-y.

## Introduction

After aneurysmal subarachnoid hemorrhage (aSAH) there is a high rate of long-term morbidity with a mortality of 20% [1]. While the blood volume in aSAH might be expected to be an independent predictor of outcome after SAH, the original qualitative grading scales are coarse and were developed for prediction of vasospasm and poorly predict outcome [2]. Due to the practical difficulties with obtaining measurements, it is only recently that quantitative measurement of blood volume based on non-contrast computerized tomography has been shown to be predictive of outcome [3, 4]. These quantitative measurements were superior to the modified Fisher scale and Hijdra score [5]. Since then, a convolutional neural network (CNN) has been developed to autonomously quantify blood volumes [4]. This has demonstrated large improvements in outcome prediction by adding blood volume to conventional models [6].

Simultaneously there has been growing interest in CSF volume as a marker of cerebral edema, specifically sulcal CSF volume, on CT scans after aSAH as a predictor of outcome. Global cerebral edema (GCE) was first described as a qualitative marker for cerebral edema within 72 h after aSAH, defined by effacement of the sulci and hemispheric cisterns with bilateral disruption of the hemispheric gray-white matter junction at the level of the semiovale [7]. This was later developed into a four-point ordinal scale called the Subarachnoid Hemorrhage Early Brain Edema Score (SEBES), which was shown to be an independent predictor of delayed cerebral ischemia (DCI) and unfavorable outcome [8]. Building further on this, the volume of CSF in the cortical sulci above the lateral ventricles – selective sulcal volume (SSV) was described in patients after aSAH [9]. A low SSV after aSAH may indicate sulcal effacement secondary to cerebral oedema and raised ICP [10]. A CNN for autonomously calculating sulcal volumes after ischemic stroke was recently repurposed for aSAH and showed that early sulcal volume defined as the lowest volume in the first 72 h after ictus, was independently predictive of outcome at discharge, but with no later outcomes presented to date [11].

Clinicians are therefore now presented with two new promising CT markers of outcome that can be accurately and rapidly measured [4, 11]. However, blood and CSF volumes might be expected to be interrelated. SSV measurements are considered indicators of cerebral edema, but they are mechanism-agnostic and reflect sulci effacement due to pressure from subarachnoid blood [12]. Therefore, it is unclear if these provide additional independent predictive value, and if so, their relative contribution to this is unknown. This complex relationship could be further complicated by pre-SAH brain and CSF volumes, both of which would be expected to be related to age [12, 13]. For equivalent sized bleeds, younger patients might be expected to have higher intracranial pressures relative to older patients with more who possess atrophic brains [12, 14]. This complex three-way interaction between blood volume, SSV and age has not been explored to date.

Therefore, the aims of this study were to: describe the relationship between the volume of blood and CSF in different compartments on baseline CT after aneurysmal subarachnoid hemorrhage, assess if they independently predict long-term outcome, and explore their interaction with age.

## Materials and methods

### Study design, setting, and participants

This study utilized data collected in the SFX-01 after subarachnoid hemorrhage (SAS) trial. This was a prospective, multicenter double-blind, placebo-controlled randomized control trial assessing the safety, pharmacokinetics, and efficacy of SFX-01 in patients aged 18–80 within 48 h of a Fisher grade 3 or 4 SAH [15]. Data was collected from the intervention and control arms of the SAS study. The trial did not identify a statistically significant difference in CSF haptoglobin levels, middle cerebral artery flow velocity or functional outcome [16].

### Data sources and measurements

#### Baseline demographics

Participant age, sex, history of hypertension, aneurysm location, Fisher score, WFNS score and time from ictus to CT scan were prospectively recorded in the study database on recruitment. The subsequent need for acute CSF diversion with external ventricular drain was also noted.

#### Analysis of CT brain scans

CT scans were performed within 48 h of ictus prior to recruitment and available for analysis. Initially the CT scans were manually reviewed by a single neurosurgical doctor and given a SEBES score. Manual segmentation of blood and CSF volumes were quantified using MIPAV (Medical Image Processing, Imaging and Visualization) v11.0 as previously described [17]. Volumes of Interest (VOIs) for blood and total CSF volume (TCV) were marked on each CT scan slice, with radiodensity thresholds set at 50–80 Hounsfield units for blood and − 5 to 20 Hounsfield units for CSF. The selective sulcal volume (SSV) was calculated by subtracting the CSF volume above the ventricles from the TCV, yielding the non-selective sulcal volume (non-SSV CSF) in the ventricles and lower sulci.

Separately, Ictus CT scan images were uploaded as NIfTI files to ITK-SNAP v4.0 for semi-automated segmentation of ventricular CSF [18], adapting a pipeline previously reported for blood segmentation [19]. A random forest classifier was trained on manually labeled data to classify each voxel in the CT scan into CSF, parenchyma, bone, or blood. A ‘speed image’ was created from CSF probability maps, and active contour evolution was used to expand manually placed seeds to fill the ventricles. The automated segmentations were reviewed by an attending neurosurgeon for accuracy, and there was good agreement between the semi-automated segmentations and the manual-derived ventricular segmentations (mean Dice score = 0.7604 ± 0.106; range = 0.365–0.928). Further information on segmentation is shown in the supplementary methodology.

#### Outcomes

Clinical outcome was available including the mRS [20, 21], and the Subarachnoid Hemorrhage Outcome Tool (SAHOT) [22] at 28, 90 and 180 days prospectively obtained by a trained and blinded assessor. Additionally, mRS at day 7 and on discharge were available. mRS was selected as the most common stroke scale, SAHOT as the only SAH specific scale.

Complete outcome data were available in 93% of patients, with no patients having missing mRS data and only two patients having missing SAHOT data. Therefore, after imputation with last one carried forward and last one carried backwards methods, complete mRS and 98% SAHOT was available for analysis. In instances where data remained missing, it was assumed to be missing at random, prompting the removal of those cases from the analysis.

### Statistical methods

The distribution of the blood and CSF volumes were reviewed using histograms and calculating the skew based on a normal distribution. A Shapiro-Wilk normality test was done to test for a normal distribution.

Univariate regressions were carried out across all the imaging variables and all the outcome measures to identify significant predictors of poor outcome. A Receiver Operating Characteristic (ROC) curve was used to assess the univariate performance of conventional SAH variables to predict day 180 mRS. The primary outcomes were mRS at day 180 and SAHOT at day 180, analyzed using ordinal regression. Secondary analyses were done using different timepoints and with outcome scores dichotomized - poor outcome was defined as: mRS 4–6 and SAHOT 5–9. The mRS cut-off was chosen based on previous dichotomizations present in the aSAH literature and the SAHOT cut-off was chosen as this the closest reflection of mRS 4–6 [6, 22].

Given the high likelihood of at least some variables being correlated principal component analysis was undertaken to investigate relationships between imaging predictor variables and WFNS.

A multivariate predictive model of outcome was developed using variables with an equal or greater importance that age and optimized with stepwise forward and backward regression based on Akaike Information Criterion (AIC) and Bayesian Information Criterion (BIC). AIC and BIC estimate prediction error in a model with lower values indicating a better model fit.

All data analysis was done in RStudio Version 4.3.2 using packages: dplyr, ggplot2, lme4m lmtest, MASS and pROC. Data analysis was guided by an expert statistician in the authorship (CW).

The manuscript was written with reference to the STrengthening the Reporting of OBservational studies in Epidemiology (STROBE) guidelines for reporting cohort studies [23].

## Results

### Patient characteristics

Between April 2016 and February 2019, 105 patients aged over 18 years consented to participation in the SAS study. Three patients had incomplete clinical outcome data and five patients’ baseline CT scans were not available as useable imaging files. Hence 97 patients were available for analysis. The baseline patient characteristics are shown in Table 1. A summary of patient outcomes is shown in Supplementary Table 1.

**Table 1:** Baseline patient characteristics

Characteristic (*N* = 97)	Summary^1^
**Age**	55 (50, 62)
**Sex**	
Female	75 (77%)
Male	22 (23%)
**Hypertension**	
No	70 (72%)
Yes	27 (28%)
**Surgical procedure**	
Clipping	22 (23%)
Coiling	73 (75%)
None performed	2 (2.1%)
**Time until scan (minutes)**	271 (131, 547)
**Fisher Grade**	
1	0 (0%)
2	0 (0%)
3	37 (38%)
4	60 (62%)
**SEBES**	
0	63 (65%)
1	15 (15%)
2	9 (9.3%)
3	7 (7.2%)
4	3 (3.1%)
**Total Blood Volume (ml)**	20 (9, 35)
**Total CSF Volume (ml)**	158 (90, 283)
**SSV Volume (ml)**	37 (20, 75)
**Ventricular CSF volume (ml)**	39 (23, 62)
**Admission WFNS**	
1	44 (45%)
2	16 (16%)
3	5 (5.2%)
4	27 (28%)
5	5 (5.2%)

^1^ Median (IQR); n (%)

SEBES, Subarachnoid Hemorrhage Early Brain Edema Score; SSV, Selective Sulcal Volume; WFNS, World Federation of Neurological Societies scale

#### Blood and CSF volume distributions

Total blood volumes had a skewed distribution across a continuous scale with a positive skew (Shapiro-Wilk normality test W = 0.927, p = < 0.001) with most patient having low blood volumes on CT imaging. Similarly, the SSV CSF (W = 0.864, *p* < 0.001), ventricular CSF volumes (W = 0.839, *p* < 0.001), and TCV (0.921, *p* < 0.001) were distributed across the continuous scale with a positive skew (Supplement Fig. 1 + 2).

### Main results

#### Univariate analysis

**Fig. 1:**
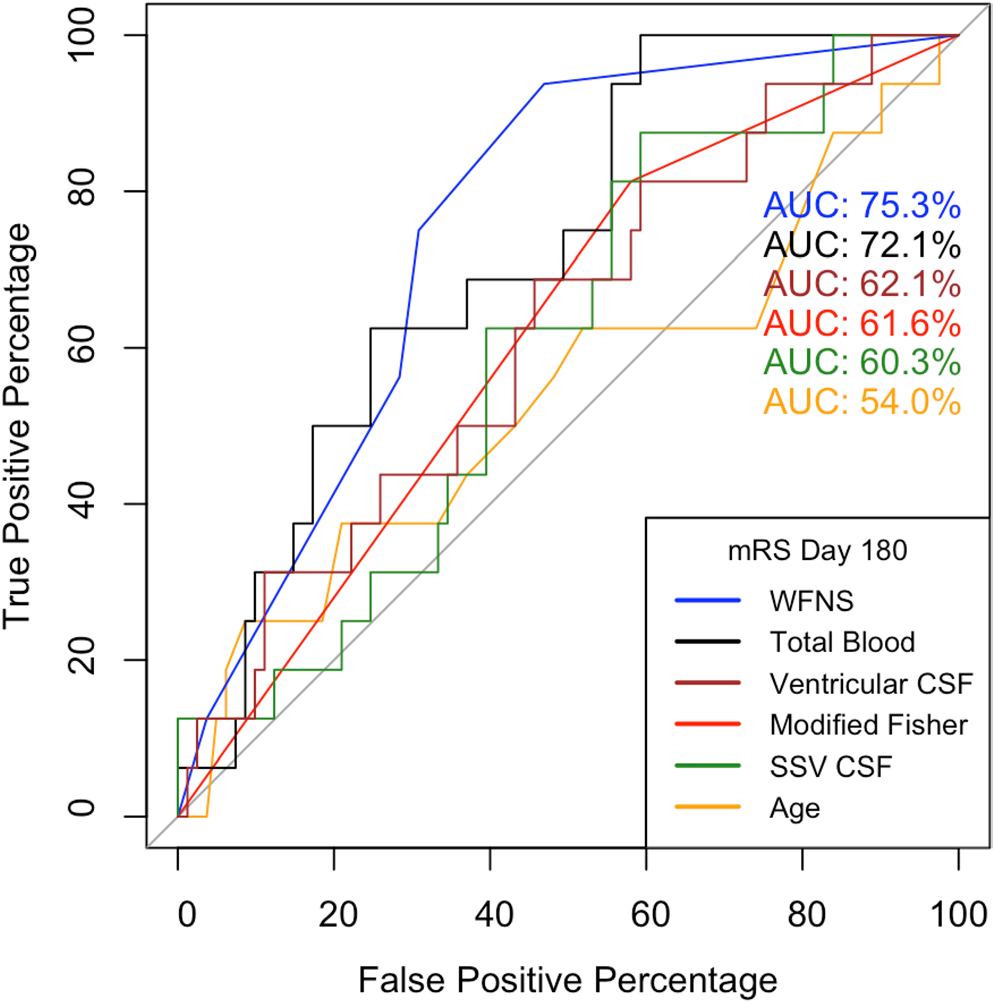
Receiver operator characteristic (ROC) curve of conventional predictors of outcome after subarachnoid hemorrrhage. mRS, modified Rankin Score; WFNS, World Federation of Neurological Societies scale; SSV, Selective Sulcal Volume

Univariate logistic regressions to predict poor outcome were performed for each of the predictive variables across all the outcome measures and are shown in Supplementary Tables 2 and 3. A ROC curve indicated that the WFNS score has the best predictive performance, while age shows the least discrimination ability to predict mRS at day 180. The TBV is the best performing imaging predictor followed by ventricular CSF volume and modified Fisher scale (Fig. 1).

A summary of ordinal regression analyses at day 180 for the key imaging predictors and WFNS are shown in Table 2.

**Table 2:** Univariate ordinal regression of long-term outcome scores

Predictor	mRS day 180^1^	SAHOT day 180^1^
WFNS	0.567 (< 0.001 ***)	0.279 (0.037 *)
Total Blood Volume	0.029 (0.004 **)	0.035 (< 0.001 ***)
SSV CSF	-0.007 (0.084)	-0.008 (0.036 *)
Ventricular CSF	0.004 (0.414)	-0.001 (0.807)

^1^Estimated coefficients and (likelihood ratio test) p-values from fitting ordinal regressions

mRS, modified Rankin Score; SAHOT, Specific Subarachnoid Hemorrhage Outcome Tool; SSV, Selective Sulcal Volume; WFNS, World Federation of Neurological Societies scale

* Indicates *p* < 0.05, ** Indicates *p* < 0.01, *** Indicates *p* < 0.001

#### Principal component analysis

**Fig. 2:**
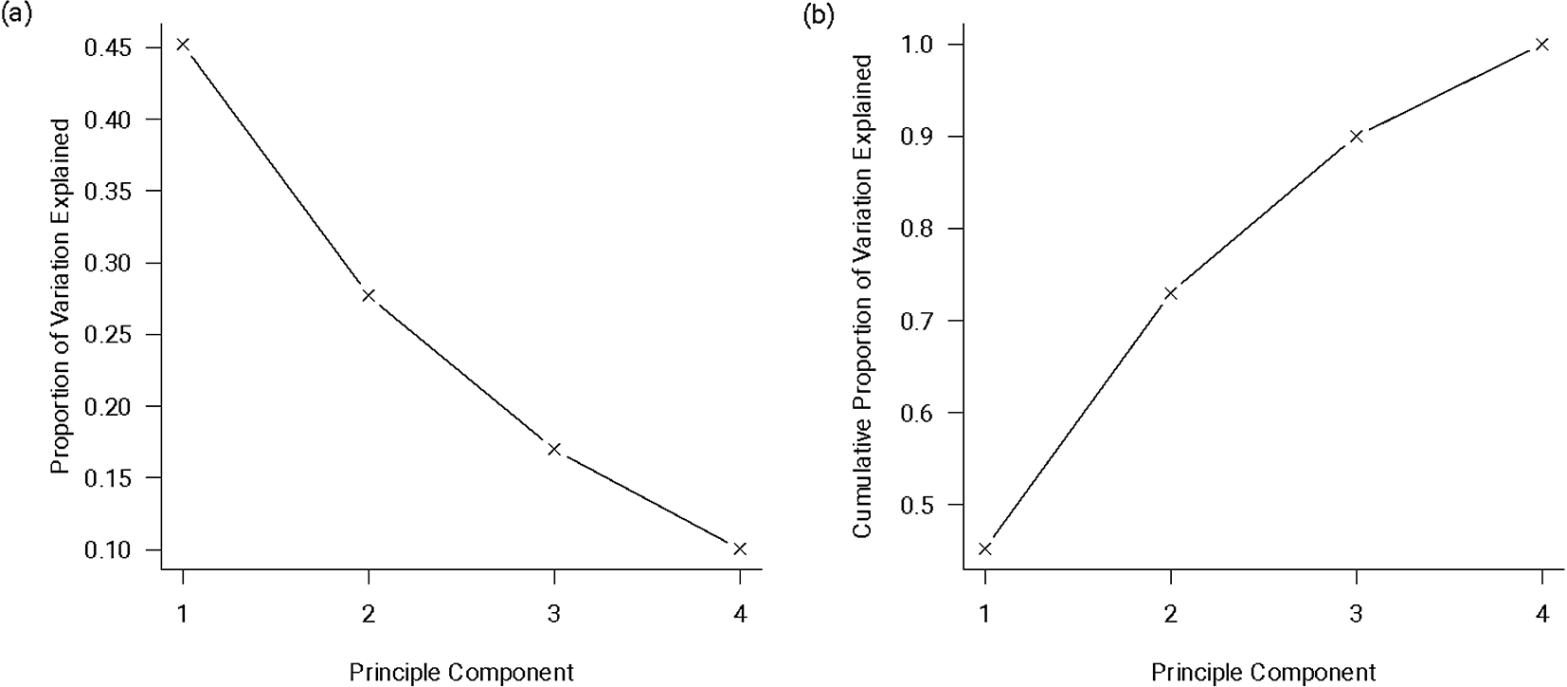
Proportional and cumulative proportion of variance from the principal component analysis

We use Principal Component Analysis (PCA) to reduce the dimensionality of the main predictor data so that we can examine the shape and structure of the data cloud of these four predictors. To this end, PCA produces a new set of orthogonal variables (i.e., the correlation values among them are 0) – principal components (PCs), each of which is a linear combination (a weighted sum) of the four standardized predictors. The standardization divides each predictor by its standard deviation and removes the choice of units, which affects the results of PCA. The PC1 represents the axis (i.e., vector) that explains the largest variance in the (standardized) data cloud, while PC2 explains the second largest, and so on and so forth (Fig. 2).

According to Table 3, PC1 has moderate to strong correlations with the four variables, having the strongest correlation with total blood volume (0.746) and the weakest with ventricular volume (-0.557). This means that PC1 is more informative of the variation of total blood than that of ventricular volume. The direction of PC1 correlation with WFNS and total blood volume is opposite to that with SSV and ventricular volume. Therefore, increasing WFNS or TBV (decreasing SSV or ventricular volume) increases the PC1 values. On the other hand, PC2 is only moderately correlated with WFNS and highly correlated with ventricular volume, indicating that the variation of ventricular volume is more closely represented by PC2. The correlations between PC3 (or PC4) and the predictors are generally weak and, thus, are probably not so informative of the patterns in the data cloud.

**Table 3:** Correlation between the principal component analysis scores and the predictors

PCs	Predictors			
	WFNS	Total Blood	SSV Volume	Ventricular Volume
PC1	0.652	0.746	-0.719	-0.557
PC2	0.624	0.314	0.341	0.711
PC3	0.218	-0.499	-0.546	0.291
PC4	0.372	-0.309	0.262	-0.316

SSV, Selective Sulcal Volume; WFNS, World Federation of Neurological Societies scale

As shown in Supplement Table 4, the PC1 coefficients all have similar magnitudes amongst the predictors, suggesting similar contributions across all four predictors. PC2 coefficients for WFNS (0.592) and ventricular volume (0.675) have substantially greater magnitudes than TBV (0.298) and SSV volume (0.324), indicating that PC2 is dominated by WFNS and ventricular volume. Since PC3 and PC4 explain less proportions of variation and are only weakly correlated with the predictors, their coefficients may not provide useful information.

The PCs were then used to predict long-term outcome using univariate ordinal regression (see Table 4). PC1 and PC2 predict mRS day 180 with similar p values to WFNS and TBV. Additionally, PC1 is highly significant for predicting SAHOT on day 180. This suggests that additional information may be provided by the PCs that the individual imaging markers alone, and that a combination of imaging predictors could result in better prediction of outcome. We, therefore, progressed with a multivariate analysis.

**Table 4:** Principal component coefficients predicting outcome

PCs	mRS day 180	SAHOT day 180
	ordinal	ordinal
PC1	0.473 (0.001) **	0.422 (0.002**)
PC2	0.563 (0.002) **	0.250 (0.134)
PC3	0.241 (0.286)	-0.0536 (0.819)
PC4	-0.044 (0.882)	-0.618 (0.038 *)

^1^Estimated coefficients and (likelihood ratio test) p-values from fitting ordinal regressions

mRS, modified Rankin Score; SAHOT, Specific Subarachnoid Hemorrhage Outcome Tool; SSV, Selective Sulcal Volume; mRS, World Federation of Neurological Societies scale

* Indicates *p* < 0.05, ** Indicates *p* < 0.01, *** Indicates *p* < 0.001

#### Multivariate regression model

A multivariate model was developed using best-subset selection for mRS and SAHOT on day 180 based on the AIC and BIC measures. All combinations of WFNS and the imaging predictors, together with sensitivity analyses using dichotomized mRS and SAHOT outcomes and log transformed predictors are displayed in Supplement Tables 5 and 6. On both AIC and BIC on the primary analysis using ordinal regression, the model with just WFNS generated the best fit for mRS and the model with just TBV the best fit for SAHOT.

### Other analyses

#### Interactions between age, CSF, and blood volume

**Fig. 3:**
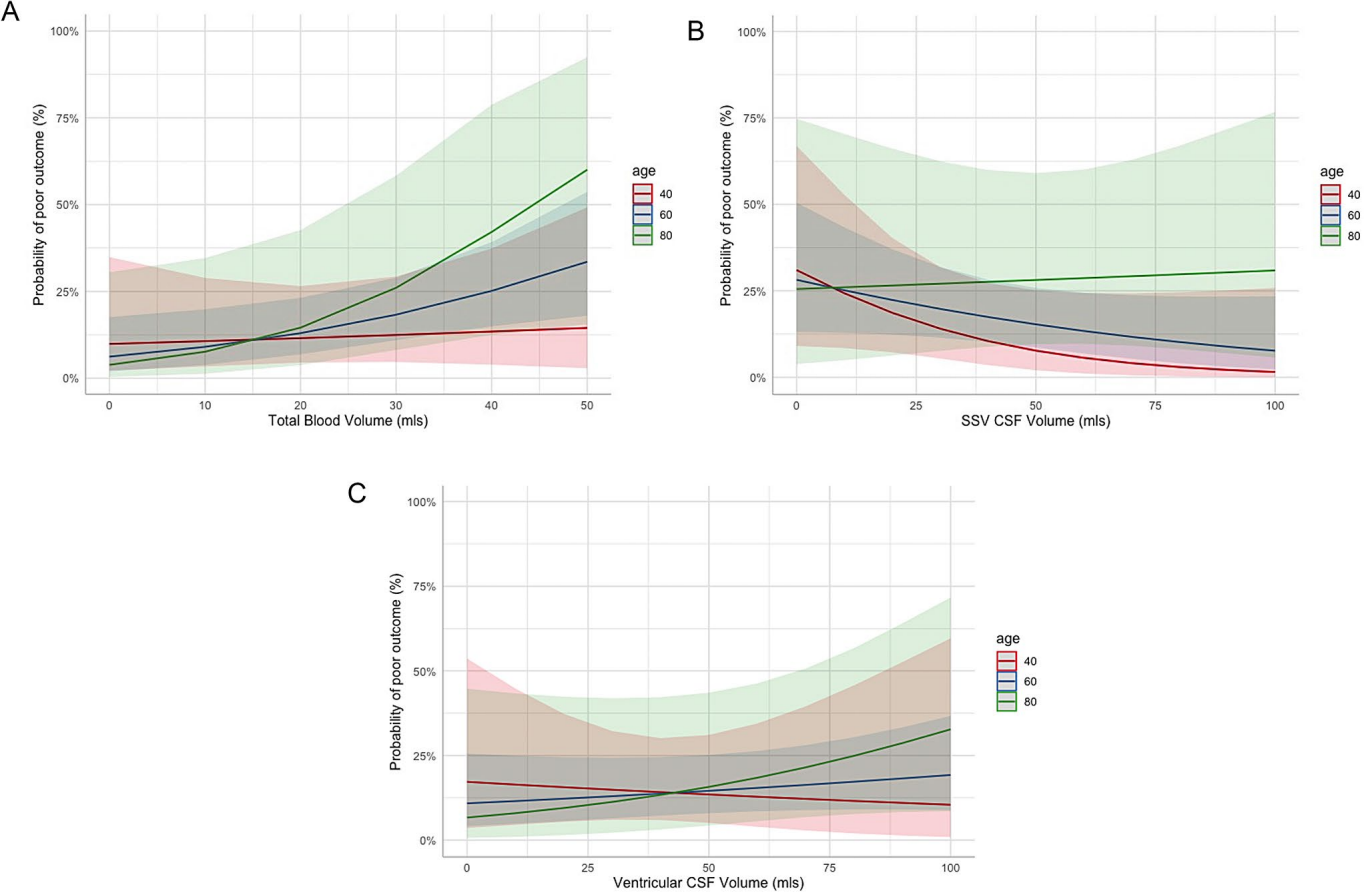
Interaction between age at 40, 60, and 80 years, and (**a**) Total Blood Volume, (**b**) SSV CSF and (**c**) Ventricular CSF Volume for prediction of mRS at day 180. SSV, Selective Sulcal Volume

Interaction plots of total blood volume, SSV CSF and ventricular CSF with age are shown in Fig. 3a-c. Although the effect sizes are large, the confidence intervals are wide due to small subgroup numbers and individual interaction variables consequently were not powered to show significant differences. However, the plots are suggestive that the effects of increasing blood volume may be greater with increasing age (Fig. 3a). SSV CSF showed a similar pattern and only in young patients does there appear to be any suggestion of the reported worse outcomes with low SSV CSF (Fig. 3b). Ventricular CSF showed a similar pattern with increasing volumes associated with worse outcome particularly in the elderly and low ventricular CSF related to worse outcome in young patients (Fig. 3c). This complex relationship is further illustrated in a three-way interaction plot between TBV, SSV CSF, and age in Fig. 3a. This suggests that in young patients low SSV CSF is related to poorer outcome irrespective of blood volume, but that in older patients this only applies to patients with low blood volume but not those with high blood volume where high SSV CSF appears related to worse outcomes.

**Fig. 4:**
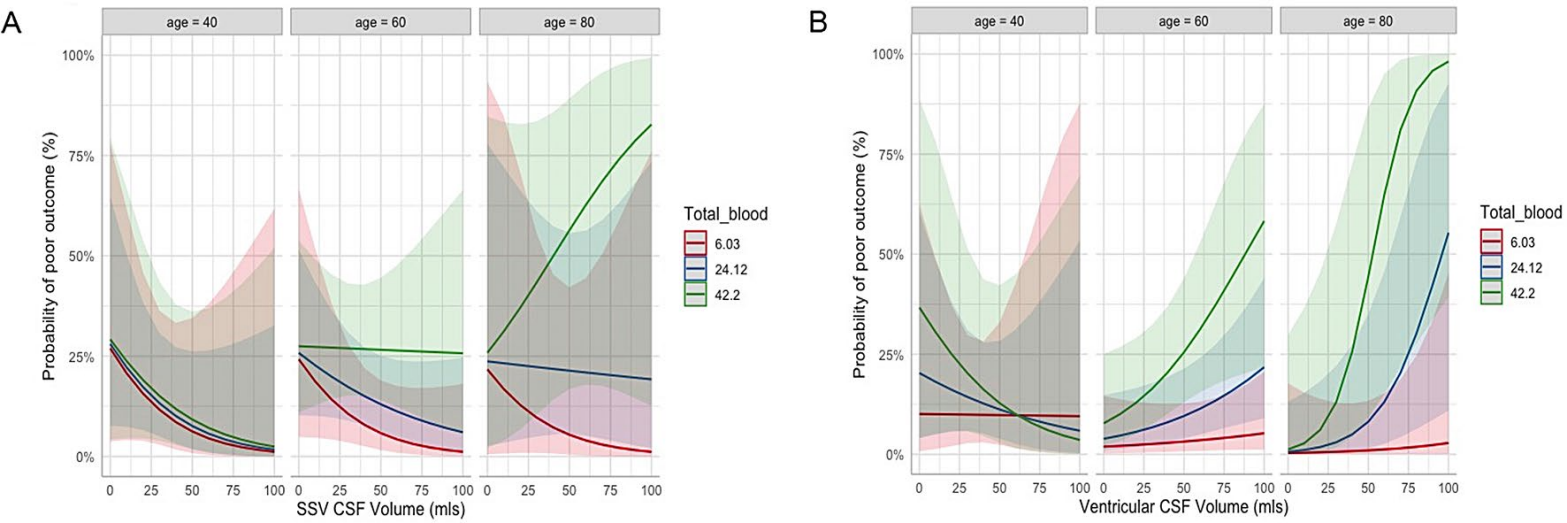
Three-way interaction between age, total blood volume at 6.03 ml, 24.12 ml and 42.2 ml, and (**a**) SSV CSF. (**b**) Ventricular CSF. Blood volumes were selected by the model as they represent lower, middle and upper thirds in the cohort. Shaded areas represent the confidence intervals of the regressions. SSV, Selective Sulcal Volume; TBV, Total Blood Volume

A three-way interaction plot between TBV, ventricular CSF and age is shown in Fig. 4b. Specific total blood volumes of 6.03 ml, 24.12 ml and 42.2 ml were chosen as they represented lower, middle, and upper thirds of the blood volumes in our cohort. This appears to show that while in young patients’ ventricular size has little influence on outcome, in older groups with higher blood load it appears progressively more associated with worse outcome.

### Sensitivity analysis

Patients were separated into quartiles based on their SSV volume on admission CT scan. At the earlier timepoint of mRS day 28, patients in the lowest SSV CSF volume quartile have significantly worse outcome. However, SSV does not reach significance at the day 180 timepoint. In addition, SSV CSF volume was dichotomized into ‘low’ <5.2 ml and ‘high’ >5.2 ml. This identified that only in the early timepoint of mRS day 28, ‘low’ SSV CSF volume predicted poor outcome (see Supplement Table 2). Additionally, the subjective categorical scale that preceded SSV quantification – SEBES - was only predictive at a few individual timepoints. In this dataset maintaining SSV as a continuous variable achieved a better, albeit still limited, predictive value than any of the published alternatives.

## Discussion

### Key results and interpretation

The aims of this study were to describe the relationship between blood and CSF volumes on baseline CT scans after aSAH, assess if they independently predict long-term outcome, and explore their interaction with age. We utilized a semi-autonomous segmentation pipeline developed in a previous paper by Street et al. to precisely measure ventricular volumes, which had high inter-rater agreement with experts [19]. In addition, the dataset was collected in a prospective cohort that was followed up for 6 months after presentation, allowing for longer-term outcomes following aSAH to be investigated.

Firstly, this study shows in a cohort of Fisher 3 and 4 aSAH patients, hemorrhaged blood volume is the most significant measured imaging predictor of poor outcome and that it outperforms all other previously reported imaging markers. The post-resuscitation WFNS remains the strongest predictor of outcome overall. Hemorrhaged blood volume also seems particularly closely linked to SAHOT. This may suggest that SAHOT is capturing a different part of the SAH injury to mRS and that TBV and WFNS may be predicting slightly different patho-physiological processes. Further investigation is necessary to better understand the specific aspects of injury captured by the mRS and SAHOT, and how these relate to the predictors.

Secondly, this study shows limited predictive value for SSV CSF at long term timepoints when using mRS. However, we have replicated previous findings that subsets of patients with low SSV CSF within 72 h have poor outcome up to a month after aSAH [11]. There are three possible explanations for this that may be working in concert. Given SSV CSF has to date only been shown to be predictive of discharge outcome in a relatively large series [11], it is therefore possible it is only weakly predictive of very early outcomes and not at all for later outcomes. Alternatively, it has been suggested that the relationship between low SSV and poor outcome is only observed in younger patient groups [11]. This is because in elderly patients the vulnerability of the brain to neurological damage with GCE is reduced due to improved compliance from brain atrophy [24]. There is some support for this theory from our interaction plots, which indicate that in patients aged 40, low SSV volumes lead to worse outcome irrespective of the blood volume. The other alternative is that, given SSV has only been shown to be predictive of outcome on scans obtained within 72 h of ictus, it is possible that baseline scans done at a median time delay of 271 min in this study were too early to observe this relationship [11]. In addition, we identified that SEBES was not a consistent predictor of outcome and had inferior performance to SSV CSF at early timepoints (supplement Table 2 + 3). This may be due to the four-point SEBES scale having insufficient granularity to identify patients with GCE in this dataset.

Thirdly, this is the first study to quantify ventricular CSF volume after SAH and relate it to other CSF imaging variables and outcome. Our hypothesis is that a higher ventricular volume on ictus CT scans predict poor outcome due to two different reasons; it can reflect the presence of hydrocephalus and raised intracranial pressure, but it can also reflect older patients, who are known to have worse prognosis [25]. However the effects of that may be countered by the fact that low ventricular volumes in young patients confer worse prognosis. While many patients with small ventricles may have a good prognosis, some young patients who present in coma without hydrocephalus or a reversible, may have particularly poor outcomes if their small ventricles are a consequence of the limited subarachnoid CSF space to accommodate the blood released, may have particularly poor outcomes. This might explain why overall little effect was seen. The alternative explanation would be that hydrocephalus is a completely reversible process and was effectively treated with CSF drainage in this series. However, there was no interaction with CSF diversion (data not presented), and it would be contrary to the wide literature showing patients with hydrocephalus after aSAH have worse outcome [26, 27].

Fourthly, in this study we explored the relationship between the main predictors, namely WFNS, TBV, SSV CSF and ventricular CSF. While developing a multivariate model for predicting long-term outcomes through ordinal regression, it was determined that single predictor models featuring WFNS for mRS and TBV for SAHOT gave optimal results. When additional predictors were added to the models, the predictors lost significance. This indicated that collinearity existed amongst the main predictors, and we performed a PCA to further investigate such collinearity among the main predictors. This showed that PC1 and PC2 explained most of the variance in the dataset. PC1 was correlated with TBV and WFNS and has roughly equal contributions from all predictors. In contrast PC2 was highly correlated and dominated by WFNS and ventricular volume. Further analysis using PCs to predict long-term outcome showed that PC1 and PC2, are significant predictors of mRS, and PC1 is highly significant for predicting SAHOT. The interpretation of these results is challenging but indicates that while several main predictors exist, the independent information they each give about a patient’s presentation and prognostic outcome is limited. Most of the prognostic information can be gained from two variables – WFNS and TBV.

In addition to the difficulties posed by correlations between variables, we have observed signs that there may be complex interactions between age, total blood volume and CSF volume. Unfortunately, the interaction analysis was underpowered, and it is not possible to draw any firm conclusions from this. The suggestion that both high TBV and high ventricular CSF have less influence on outcome in young patients compared to old patients is also interesting and merits further investigation. We had hypothesized that a higher total blood volumes would lead to higher intracranial pressure (ICP) in younger patients and therefore have a greater influence on outcome than in the elderly. However, the interaction plots make this very unlikely and if anything, the reverse likely to be true indicating that the blood itself is harmful rather than the ICP rise it causes and that the elderly brain is more vulnerable to this blood-mediated injury than a young brain.

### Strengths and limitations

The use of a prospectively collected study population from a randomized control trial means that the sample is highly phenotyped with very little missing data. This contrasts with many previous studies predicting outcome that are from retrospective cohorts with considerable missing data requiring data imputation [6, 11]. Additionally, it meant later 180-day outcomes were available when much previous research has been limited to earlier time points such as discharge and few have followed up longer than 3 months [11]. The study used a robust methodology for collecting blood and CSF volumes. Manual segmentation was used to collect volumes within the irregular dimensions of the sulci, whereas in the regular shape of the ventricles a semi-autonomous segmentation method was used with high Dice scores. In addition, the study collected CSF volumes in multiple forms to investigate previous findings in the literature, including calculating SSV CSF, categorizing patients into a ‘low’ SSV group, and calculating SEBES scores for each scan.

A limitation of this study population is that it was restricted to Fisher 3 and 4 patients. Results are therefore potentially not generalizable to all SAH patients. However, in the modern era with early CT scanning the relative frequency of Fisher 1 and 2 SAH is relatively low [28] and in our own center represents only 29% of aneurysmal SAH that present acutely. The patients in the study were randomized between SFX-01 and placebo which could have further influenced our results. However, this is unlikely as the study failed to meet its primary endpoints and accounting for drug allocation did not significantly alter any of the analyses.

## Conclusions

This study has demonstrated that total blood volume appears to correlate better with outcome after aSAH compared to the traditional modified Fisher scale. The SSV CSF and ventricular volume have limited predictive value for long term outcome in this series. However, there are significant correlations between imaging variables and possible complex interactions that merit further investigation with larger datasets.

## Electronic supplementary material

Below is the link to the electronic supplementary material.

Supplementary Material 1

## Data Availability

Data will be made available on reasonable request.

## Abbreviations

AIC: Akaike Information Criterion
aSAH: aneurysmal Subarachnoid Hemorrhage
CNN: Convolutional Neural Network
BIC: Bayesian Information Criterion
DCI: Delayed Cerebral Ischemia
GCE: Global Cerebral Edema
MIPAV: Medical Image Processing, Imaging and Visualization
PCA: Principal Component Analysis
PC: Principal Components
SAHOT: Specific Subarachnoid Hemorrhage Outcome Tool
ROI: Receiver Operator Characteristic
SAS: SFX-01 after subarachnoid hemorrhage trial
SEBES: Subarachnoid Hemorrhage Early Brain Edema Score
SSV: Selective Sulcal Volume
TBV: Total Blood Volume
TCV: Total CSF Volume
VOI: Volume Of Interest
WFNS: World Federation of Neurological Societies scale
