# Supplementary material for: Quantification of blood and CSF volume to predict outcome after aneurysmal subarachnoid hemorrhage
Source: Neurosurg Rev. 2024 Oct 8;47(1):752. doi: 10.1007/s10143-024-03001-y (PMC11461592; doi:10.1007/s10143-024-03001-y)
Supplement: Supplementary file 1 — Supplementary Material 1 [file 10143_2024_3001_MOESM1_ESM.docx]

# **Supplement**

# **Supplementary methods**

## Manual segmentation

Ictus CT scan images were uploaded as DICOM files to MIPAV for manual segmentation. The slices of images were made contiguous by using the formula:

$Slice thickness =$ $image position between slices$ **x cos (**$gantry/(detector tilt)$**)**

On each slice of the CT scan, Volumes of Interest (VOIs) were demarcated for blood and the radiodensity threshold was set to 50-80 Hounsfield units. Based on the voxel sum intensities in the VOIs, the total number of voxels contained and the total volume of VOIs, the total blood volumes (TBV) was generated using the formula:

$Volume (ml) = (volume of VOIs x ((voxel sum intensities)/( number of voxels )))$

A similar approach was repeated for total CSF volume (TCV). Following demarcation of the VOIs the radiodensity threshold was set to -5, 20 Hounsfield units (see Fig. 1). The Selective Sulcal Volume (SSV) was defined as the sulci above the level of the semiovale. This was calculated by measuring the CSF spaces above the highest slice on which the ventricles were visible to give the SSV CSF. This was subsequently subtracted from the Total CSF Volume (TCV) to give the non-selective sulcal volume of CSF (non-SSV CSF), this comprised on CSF present in the ventricles and in sulci below the semiovale.


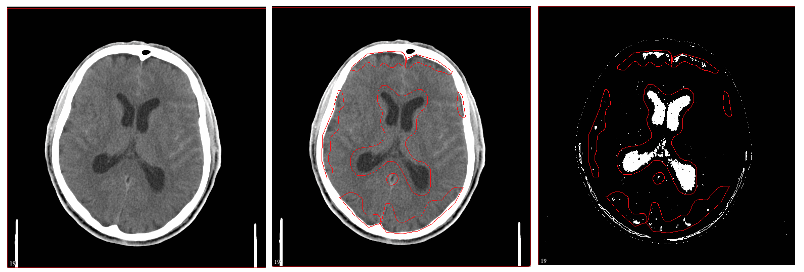


A

B

C

**Fig 1. Manual Segmentation of CSF volumes.**

(A) Ictus CT scans were obtained for each patient in the study cohort and uploaded as DICOM files to MIPAV imaging platform. (B) Volumes of interest (VOI) were manually drawn around CSF spaces for each slice of the CT scan. (C) The images were thresholded at a range of Hounsfield units, for CSF this was -5, 20. Multiplying the volume of VOIs by the sum intensities allowed for the extraction of CSF volume.

## Semi-automated segmentation

Ictus CT scan images were separately converted to NIfTI format using the open source tool dcm2niix (https://github.com/rordenlab/dcm2niix), and uploaded as NIfTI files to ITK-SNAP v4.0 for semi-automated segmentation of ventricular CSF volumes. Briefly, on each scan, a random forest classifier was trained from manually labelled training data to classify the tissue present at each voxel into classes of CSF, parenchyma, bone, or blood. A ‘speed image’ was generated from probability maps for the CSF class, and active contour evolution was subsequently run over manually placed seeds and allowed to expand to fill the ventricles.

To ensure maximal alignment with manually labelled segmentations used elsewhere in this paper, we loaded the semi-automated ventricular volumes into MATLAB (R2019b, Mathworks Ltd., Cambridge, MA) and identified connected regions within the manual segmentations that overlapped with the semi-automated segmentations. Regions containing any overlap with the semi-automated segmentation were kept, while regions with no overlap were discarded as non-ventricular, resulting in a set of ‘manual-derived’ ventricular volumes on which all further analysis was performed (see Fig. 2).


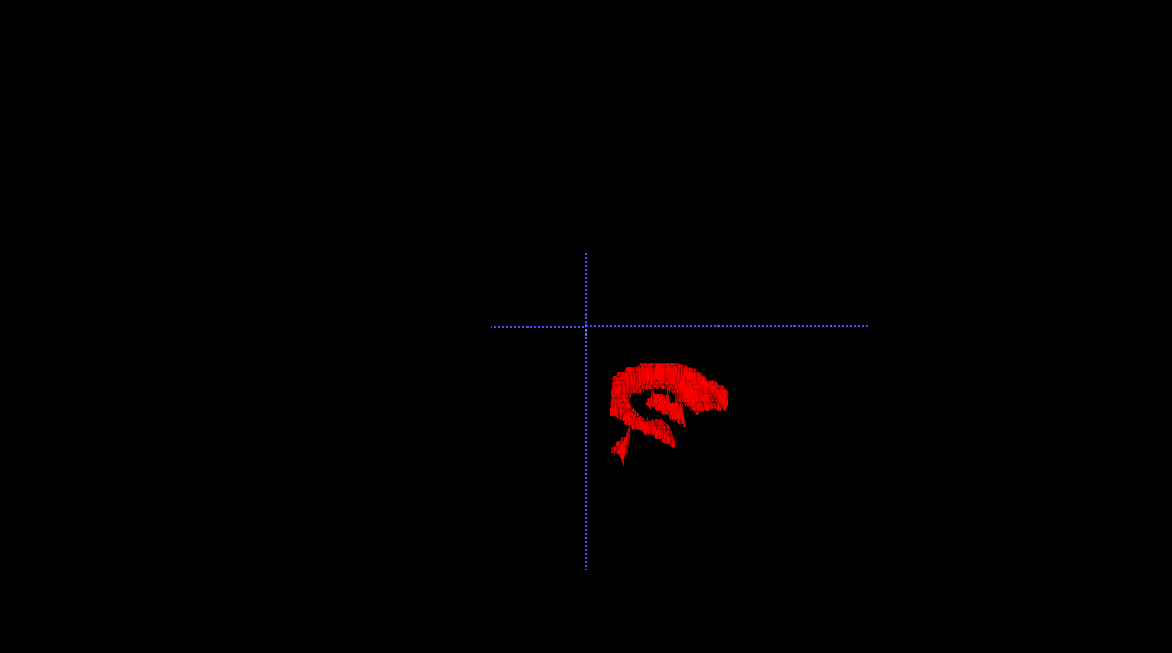

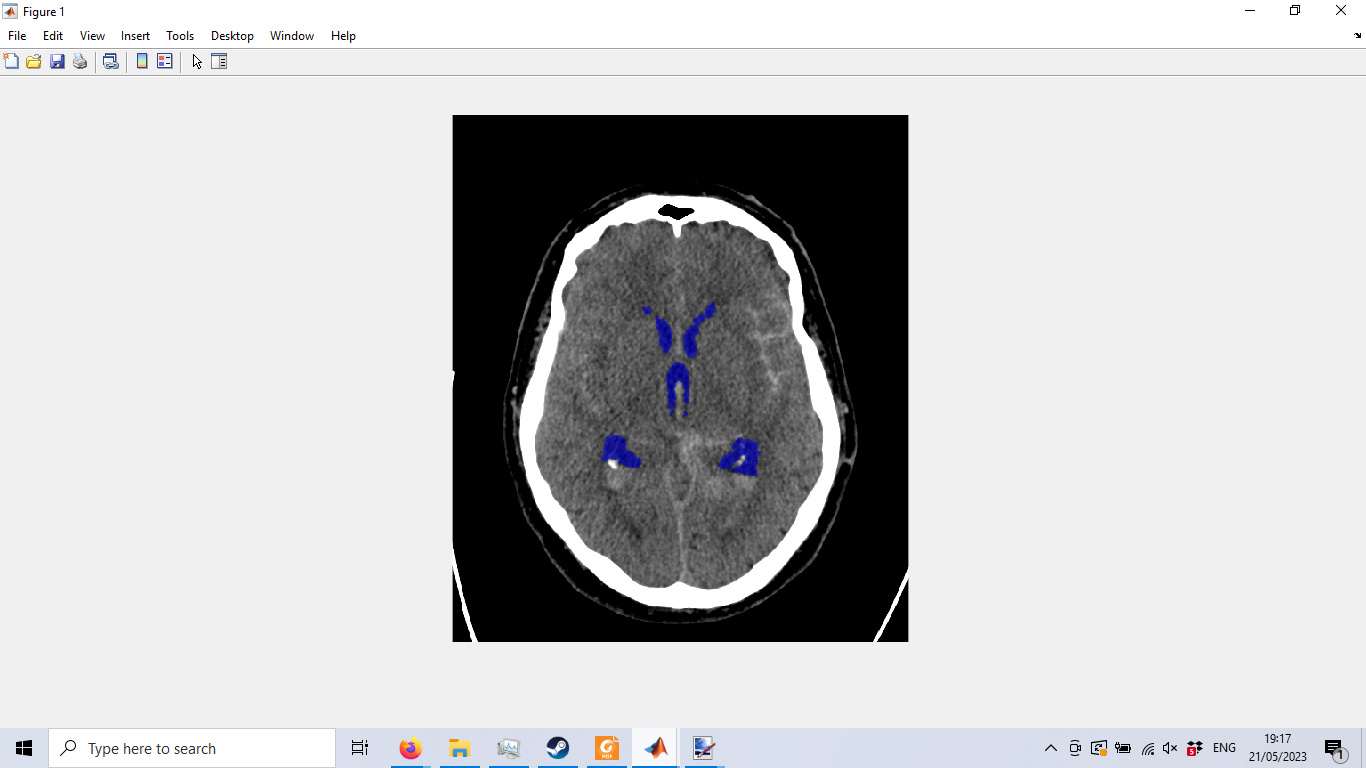

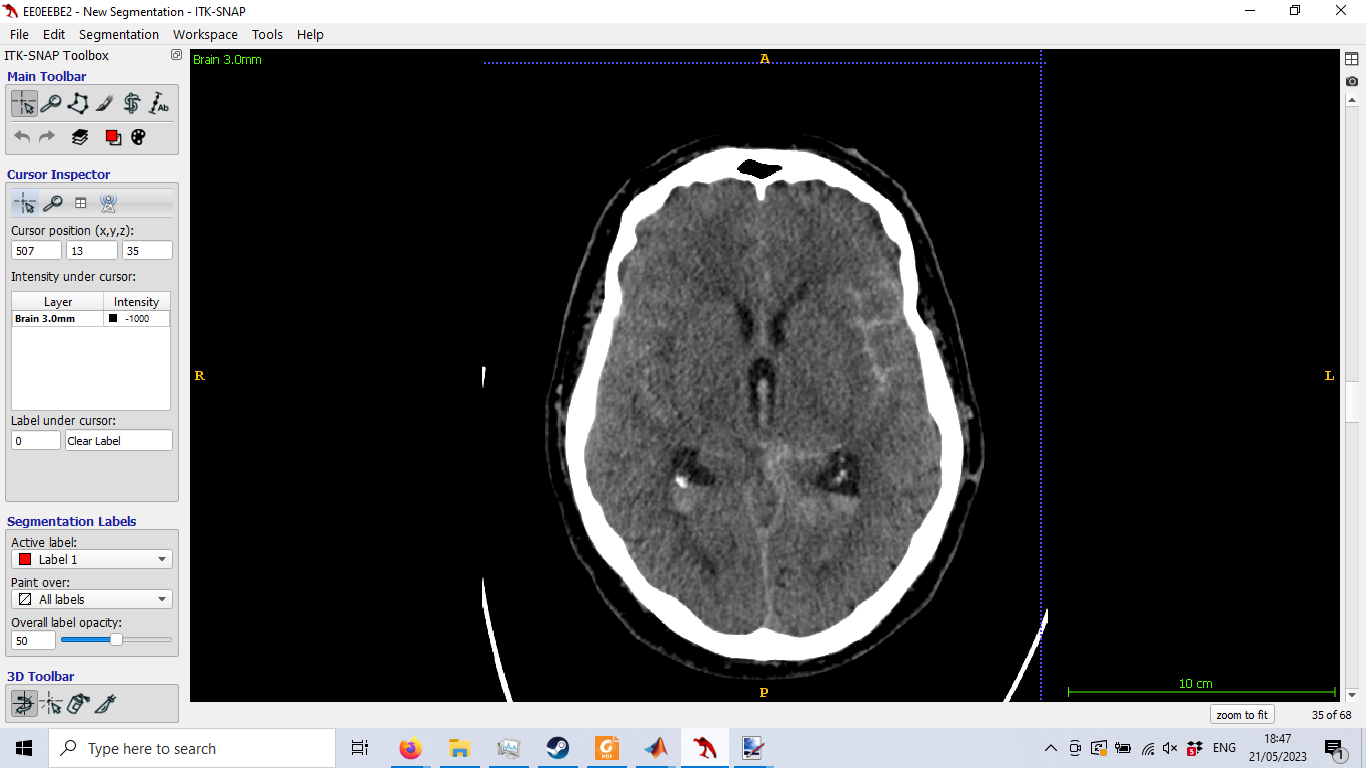


A

B

C

**Fig. 2. Semi-autonomous Segmentation.**

(A) Ictus CT scans for each patient were uploaded as NIfTI files to ITK-SNAP. (B) A random forest classifier was trained by manually labelling a selection of slices of each scan to identify CSF, parenchyma, bone, and blood. This created probability maps for CSF, which were used to collect ventricular volume by placing seeds in each ventricle and allowing them to expand. (C) The final 3D segmentation of the ventricular system allowed for the extraction of ventricle CSF volume.

All manual and manual-derived volumes were thresholded to include voxels with Hounsfield Units in the range of [-5, 20]. Overall, we saw good agreement between the semi-automated segmentations and the manual-derived ventricular segmentations (mean Dice score = 0.7604 ± 0.106; range = 0.365-0.928). Semi-automated segmentations were performed by two operators (J.B., J.S.S.), with good inter-operator agreement between demonstrated over a set of 10 training scans (median Dice score = 0.8196 ± 0.0729; ICC of segment volumes (Boers et al 2014)^3^ = 0.9909, 95% CI 0.9661-0.9977). Semi-automated segmentations were also reviewed by an attending neurosurgeon to ensure accuracy.

# **Supplementary Tables**

**Table 1. Distribution of 97 patients amongst all the outcome measures.**

| **Outcome** | **Discharge** | **Day 7** | **Day 28** | **Day 90** | **Day 180** |
| --- | --- | --- | --- | --- | --- |
| mRS  0  1  2  3  4  5  6 | 2  4  25  24  23  17  2 | 1  2  12  28  24  28  2 | 1  10  29  24  16  14  3 | 5  16  41  20  7  3  5 | 13  32  25  11  8  2  6 |
| SAHOT  1  2  3  4  5  6  7  8  9 |  |  | 2  12  11  19  18  16  6  8  3 | 6  18  17  18  16  10  3  2  5 | 17  14  18  12  13  8  4  3  6 |
| ^1^n (%)  mRS, modified Rankin Scale; SAHOT, Specific Subarachnoid Hemorrhage Outcome Tool. | | | | | |

**Table 2. Univariate regressions for modified Rankin Score analyses.**

| **Predictor** | **mRS D7^1^** | **mRS discharge^1^** | **mRS D28^1^** | **mRS D90^1^** | **mRS D180^1^** | **mRS D28 7-point^2^** | **mRS D90 7-point^2^** | **mRS D180 7-point^2^** |
| --- | --- | --- | --- | --- | --- | --- | --- | --- |
| Age | 0.039 (*0.043**) | 0.039 (*0.*0532) | 0.017 (*0.374*) | 0.007 (*0.778*) | 0.017 (*0.486*) | 0.013 (0.305) | 0.005 (0.695) | 0.006 (0.695) |
| Sex (Male) | -1.280  (*0.013**) | -1.197  (*0.032**) | -0.406  (*0.449*) | -0.740  (*0.356*) | -0.284  (*0.682*) | -0.660 (0.044*) | -0.552 (0.094) | -0.398 (0.302) |
| Hypertension | 0.416  (*0.371*) | 0.064  (*0.888*) | 0.043  (*0.929*) | -0.070 (*0.913*) | 0.539  (*0.349*) | -0.066 (0.832) | 0.139 (0.655) | 0.425 (0.239) |
| Aneurysm location (Posterior circulation) | 0.962  (*0.124*) | 1.151  (*0.053*) | 1.313  (*0.024**) | 0.934  (*.0.166*) | 0.824  (*0.217*) | 0.725 (0.057) | 0.050 (0.897) | 0.763 (0.087) |
| Surgical procedure  (Coiling) | -0.735  (*0.153*) | -0.360  (*0.461*) | 0.110  (*0.833*) | 0.117  (*0.868*) | -0.225  (*0.726*) | 0.105 (0.753) | 0.170 (0.608) | 0.300 (0.437) |
| Admission WFNS | 1.312  (*<0.001****) | 1.231  (*<0.001****) | 0.970  (*<0.001****) | 0.710  (*0.002***) | 0.652  (*0.002***) | 0.559 (<0.001***) | 0.366 (<0.001***) | 0.567 (<0.001***) |
| Fisher grade | 1.190  (*0.006***) | 1.336  (*0.004***) | 1.442  (*0.005***) | 0.616  (*0.325*) | 1.143  (*0.092*) | 0.716 (0.011*) | 0.355 (0.213) | 0.551 (0.096) |
| SSV blood | 0.009  (0.861) | 0.033  (*0.524*) | 0.021  (*0.689*) | 0.034  (*0.573*) | 0  (*0.995*) | 0.020 (0.567) | 0.031 (0.381) | 0.039 (0.342) |
| Non-SSV blood | 0.084  (*<0.001****) | 0.069  (*<0.001****) | 0.043  (*0.003)* | 0.042  (*0.026**) | 0.045  (*0.007***) | 0.034 (<0.001***) | 0.023 (0.008**) | 0.031 (0.002**) |
| Total blood | 0.069  (<*0.001****) | 0.063  (<*0.001****) | 0.041  (*0.002***) | 0.032  (*0.034**) | 0.036  (*0.015**) | 0.032 (<0.001***) | 0.021 (0.005**) | 0.029 (0.004**) |
| SEBES | 0.226  (*0.249*) | 0.258  (*0.172*) | 0.275  (*0.148*) | 0.521  (*0.018**) | 0.123  (*0.600*) | 0.285 (0.023*) | 0.289 (0.021*) | 0.236 (0.107) |
| SSV CSF | -0.005  (0.272) | -0.006  (0.181) | -0.013  (0.026*) | -0.017  (0.078) | -0.013  (0.129) | -0.007 (0.022*) | -0.007 (0.028*) | -0.007 (0.084) |
| SSV CSF ‘lowest quartile’ | 1.340  (0.031*) | 1.281  (0.035*) | 1.825  (0.007**) | 1.107  (0.215) | 0.833  (0.364) | 1.187 (0.002**) | 0.665 (0.084) | 0.857 (0.060) |
| SSV CSF <5.2ml | 1.126  (0.032*) | 0.811  (0.090) | 1.142  (0.019*) | 0.506  (0.405) | 0.017  (0.979) | 0.872 (0.006**) | 0.324 (0.313) | 0.567 (0.129) |
| Non-SSV CSF | 0.001  (0.557) | 0.001  (*0.358*) | 0.0*02*  *(0.383*) | 0  (*0.931*) | 0.002  (*0.366*) | 0.001 (0.64) | 0 (0.766) | 0 (0.608) |
| Ventricular CSF | 0.005  (0.382) | 0.009  (0.102) | 0.008  (0.106) | 0.003  (0.632) | 0.009  (0.148) | 0.005 (0.106) | 0.002 (0.505) | 0.004 (0.414) |
| Non-SSV, non-ventricular CSF | 0.001  (0.752) | 0.001  (0.734) | 0.001  (0.775) | -0.001  (0.891) | 0.001  (0.673) | 0 (0.823) | 0 (0.965) | 0 (0.76) |
| Total CSF | 0  (*0.936)* | 0.003  (*0.800*) | 0  (0.922) | -0.001  (*0.593*) | 0  (*0.859)*) | 0 (0.695) | 0 0.623 | 0 (0.79) |
| mRS, modified Rankin Scale; WFNS, World Federation of Neurological Societies scale; SSV, Selective Sulcal Volume.  ** Indicates p < 0.05, ** Indicates p < 0.01, *** Indicates p < 0.001*  *^1^ Indicates dichotomized outcome analyzed with logistic regression.*  *^2^ Indicates ordinal outcome analyzed with ordinal regression.* | | | | | | | | |

**Table 3. Univariate regressions for Subarachnoid Hemorrhage Outcome Tool analyses.**

| **Predictor** | **SAHOT D28^1^** | **SAHOT D90^1^** | **SAHOT D180^1^** | **SAHOT D28 9-point scale^2^** | **SAHOT D90 9-point scale^2^** | **SAHOT D180^1^ 9-point scale^2^** | **SAHOT D180 Raw^3^** |
| --- | --- | --- | --- | --- | --- | --- | --- |
| Age | -0.016 (*0.395*) | -0.029 (*0.131*) | -0.030 (*0.120*) | -0.006 (0.73) | -0.024 (0.197) | -0.014  (0.494) | -0.300  (0) |
| Sex (Male) | 0.180  (*0.719*) | 0.011  (*0.983*) | 0.187  (*0.714*) | -0.389 (0.424) | -0.400 (0.425) | -0.483  (0.401) | 0.264  (0.967) |
| Hypertension | 0.441  (*0.348*) | 0.475  (*0.310*) | 0.673  (*0.155*) | 0.410 (0.364) | 0.640 (0.169) | 1.050  (0.048*) | 13.019  (0.028*) |
| Aneurysm location (Posterior circulation) | 0.251  (*0.661*) | -0.649  (*0.301*) | 0.845  (*0.123*) | 0.628 (0.259) | 0.136 (0.814) | 0.644  (0.332) | 14.027  (0.055) |
| Surgical procedure  (Coiling) | 0.255  (*0.602*) | -0.366  (*0.458*) | -0.304  (*0.543*) | 0.044 (0.927) | -0.363 (0.465) | -0.243  (0.672) | -6.615  (0.299) |
| Admission WFNS | 0.415  (*0.009***) | 0.334  (*0.031**) | 0.245  (*0.114*) | 0.611 (<0.001***) | 0.495 (<0.001***) | 0.279  (0.037*) | 3.698  (0.055) |
| Fisher grade | 0.600  (*0.160*) | 0.717  (*0.115*) | 1.183  (*0.017**) | 0.941 (0.022*) | 0.700 (0.101) | 1.105  (0.023*) | 7.442  (0.176) |
| SSV blood | 0.117  (*0.114*) | 0.169  (*0.027**) | 0.063  (*0.234*) | 0.069 (0.174) | 0.091 (0.080) | 0.082  (0.166) | 0.531  (0.470) |
| Non-SSV blood | 0.036  (*0.013**) | 0.034  (*0.014**) | 0.037  (*0.010**) | 0.035 (0.005) | 0.037 (0.003**) | 0.053  (<0.001***) | 0.503  (0.002**) |
| Total blood | 0.036  (*0.007***) | 0.0.36  (*0.004***) | 0.034  (*0.006***) | 0.033 (0.003**) | 0.035 (0.002**) | 0.035  (<0.001***) | 0.490  (0.001**) |
| SEBES | 0.423  (*0.046**) | 0.371  (*0.054*) | 0.198  (*0.300*) | 0.510 (0.004**) | 0.414 (0.026*) | 0.460  (0.031*) | 2.156  (0.4) |
| SSV CSF | -0.012  (0.021*) | -0.012  (0.044*) | -0.012  (0.048*) | -0.011 (0.014*) | -0.010 (0.022*) | -0.008  (0.036*) | -0.087  (0.145) |
| SSV CSF ‘lowest quartile’ | 1.322  (0.032*) | 0.770  (0.242) | 1.222  (0.076) | 1.460 (0.009**) | 1.069 (0.066) | 1.205  (0.075) | 10.449 (0.171) |
| SSV CSF <5.2ml | 0.721  (0.144) | -0.022  (0.963) | 0.3992  (0.411) | 0.853 (0.064) | 0.459 (0.338) | 0.587  (0.285) | 1.875  (0.767) |
| Non-SSV CSF | -0.002  (0.386) | -0.001  (*0.660*) | 0  (*0.875*) | 0 (0.929) | 0 (0.826) | -0.001  (0.822) | -0.005  (0.844) |
| Ventricular CSF | 0.001  (0.776) | -0.002  (0.669) | -0.003  (0.547) | 0.005 (0.333) | 0 (0.909) | -0.001  (0.807) | -0.035  (0.587) |
| Non-SSV, non-ventricular CSF | -0.003  (0.200) | -0.001  (0.772) | 0  (0.916) | -0.002 (0.537) | 0 (0.728) | 0  (0.989) | 0  (0.979) |
| Total CSF | 0.002  (*0.151*) | -0.002  (*0.312*) | -0.001  (*0.433*) | -0.001 (0.391) | -0.001 (0.366) | -0.001  (0.432) | -0.012  (0.534) |
| SAHOT, Subarachnoid Hemorrhage Outcome Tool; WFNS, World Federation of Neurological Societies scale; SSV, Selective Sulcal Volume.  ** Indicates p < 0.05, ** Indicates p < 0.01, *** Indicates p < 0.001*  *^1^ Indicates dichotomized outcome analyzed with logistic regression.*  *^2^ Indicates ordinal outcome analyzed with ordinal regression.*  *^3^ Indicates continuous outcome analyzed with linear regression.* | | | | | | | |

**Table 4. Principal component coefficients across predictors**

| **PCs** | **Predictors** | | | |
| --- | --- | --- | --- | --- |
|  | **WFNS** | **Total Blood** | **SSV Volume** | **Ventricular Volume** |
| PC1 | 0.485 | 0.555 | -0.535 | -0.414 |
| PC2 | 0.592 | 0.298 | 0.324 | 0.675 |
| PC3 | 0.264 | -0.605 | -0.662 | 0.353 |
| PC4 | 0.587 | -0.486 | 0.413 | -0.498 |
| SSV, Selective Sulcal Volume; WFNS, World Federation of Neurological Societies scale. | | | | |

**Table 5. All possible combinations of the four main predictors for mRS day 180.**

| **mRS180** | | | | | | |
| --- | --- | --- | --- | --- | --- | --- |
| **Model** | ***Dichotomized*** | | ***Ordinal*** | | ***Ordinal + log transformed predictors*** | |
|  | ***AIC*** | ***BIC*** | ***AIC*** | ***BIC*** | ***AIC*** | ***BIC*** |
| WFNS | 80.10589 | 85.25531 | 324.1408 | 342.1638 | 324.1408 | 342.1638 |
| Total Blood | 84.81958 | 89.96900 | 333.6260 | 351.6490 | 337.4437 | 355.4667 |
| WFNS + Total Blood | 80.58636 | 88.31049 | 324.8266 | 345.4242 | 325.9715 | 346.5692 |
| SSV CSF Volume | 87.97201 | 93.12143 | 338.7649 | 356.7879 | 337.8491 | 355.8721 |
| WFNS + SSV CSF Volume | 81.66636 | 89.39050 | 325.5705 | 346.1682 | 326.0069 | 346.6046 |
| Total Blood + SSV CSF Volume | 85.42714 | 93.15128 | 334.2201 | 354.8178 | 337.0182 | 357.6158 |
| WFNS + Total Blood + SSV CSF Volume | 82.30663 | 92.60547 | 326.4169 | 349.5893 | 327.8560 | 351.0284 |
| Ventricular CSF Volume | 88.89182 | 94.04124 | 341.0886 | 359.1116 | 340.4367 | 358.4597 |
| WFNS + Ventricular CSF Volume | 80.09875 | 87.82288 | 325.7733 | 346.3710 | 323.8668 | 344.4644 |
| Total Blood + Ventricular CSF Volume | 82.15456 | 89.87869 | 333.0881 | 353.6858 | 335.8490 | 356.4467 |
| WFNS + Total Blood + Ventricular.CSF Volume | 78.99123 | 89.29008 | 325.8405 | 349.0129 | 325.0402 | 348.2126 |
| SSV CSF Volume + Ventricular CSF Volume | 85.06317 | 92.78730 | 338.5390 | 359.1367 | 335.1473 | 355.7450 |
| WFNS + SSV CSF Volume + Ventricular CSF Volume | 80.34986 | 90.64871 | 326.6014 | 349.7738 | 324.5308 | 347.7032 |
| Total Blood + SSV CSF Volume + Ventricular CSF Volume | 80.06718 | 90.36603 | 331.9915 | 355.1639 | 331.7119 | 354.8843 |
| WFNS + Total Blood + SSV CSF Volume + Ventricular CSF Volume | 79.21571 | 92.08926 | 326.6222 | 352.3693 | 325.5154 | 351.2625 |

Note: the lower AIC and BIC for each version of the mRS180 are highlighted

**Table 6. All possible combinations of the four main predictors for SAHOT day 180**

| **SAHOT180** | | | | | | |
| --- | --- | --- | --- | --- | --- | --- |
| **Model** | ***Dichotomized*** | | ***Ordinal*** | | ***Ordinal + log transformed predictors*** | |
|  | ***AIC*** | ***BIC*** | ***AIC*** | ***BIC*** | ***AIC*** | ***BIC*** |
| WFNS | 124.1890 | 129.2967 | 432.0659 | 460.3878 | 432.0659 | 460.3878 |
| Total Blood | 118.6256 | 123.7334 | 424.1260 | 452.4478 | 427.9073 | 456.2291 |
| WFNS + Total Blood | 120.5260 | 128.1876 | 425.9941 | 456.8906 | 429.1848 | 460.0813 |
| SSV CSF Volume | 122.0236 | 127.1313 | 432.0290 | 460.3508 | 431.6294 | 459.9512 |
| WFNS + SSV CSF Volume | 123.1669 | 130.8286 | 431.1430 | 462.0395 | 431.5581 | 462.4546 |
| Total Blood + SSV CSF Volume | 118.2534 | 125.9150 | 424.5125 | 455.4090 | 427.5035 | 458.4000 |
| WFNS + Total Blood + SSV CSF Volume | 120.2469 | 130.4625 | 426.4495 | 459.9208 | 429.2881 | 462.7594 |
| Ventricular CSF Volume | 126.3281 | 131.4359 | 436.3775 | 464.6993 | 436.2584 | 464.5802 |
| WFNS + Ventricular CSF Volume | 125.7326 | 133.3942 | 433.9669 | 464.8634 | 433.7583 | 464.6549 |
| Total Blood + Ventricular CSF Volume | 120.6218 | 128.2835 | 425.6310 | 456.5275 | 428.0201 | 458.9166 |
| WFNS + Total Blood + Ventricular CSF Volume | 122.5259 | 132.7414 | 427.5799 | 461.0512 | 429.4909 | 462.9621 |
| SSV CSF Volume + Ventricular CSF Volume | 123.9486 | 131.6102 | 433.3566 | 464.2531 | 430.7125 | 461.6090 |
| WFNS + SSV CSF Volume + Ventricular CSF Volume | 125.1582 | 135.3738 | 432.7899 | 466.2612 | 431.1844 | 464.6556 |
| Total Blood + SSV CSF Volume + Ventricular CSF Volume | 119.8215 | 130.0371 | 424.5745 | 458.0458 | 424.0916 | 457.5628 |
| WFNS + Total Blood + SSV CSF Volume + Ventricular CSF Volume | 121.7449 | 134.5143 | 426.5628 | 462.6088 | 426.0766 | 462.1225 |

Note: the lower AIC and BIC for each version of the SAHOT 180 are highlighted

# **Supplementary Figures**


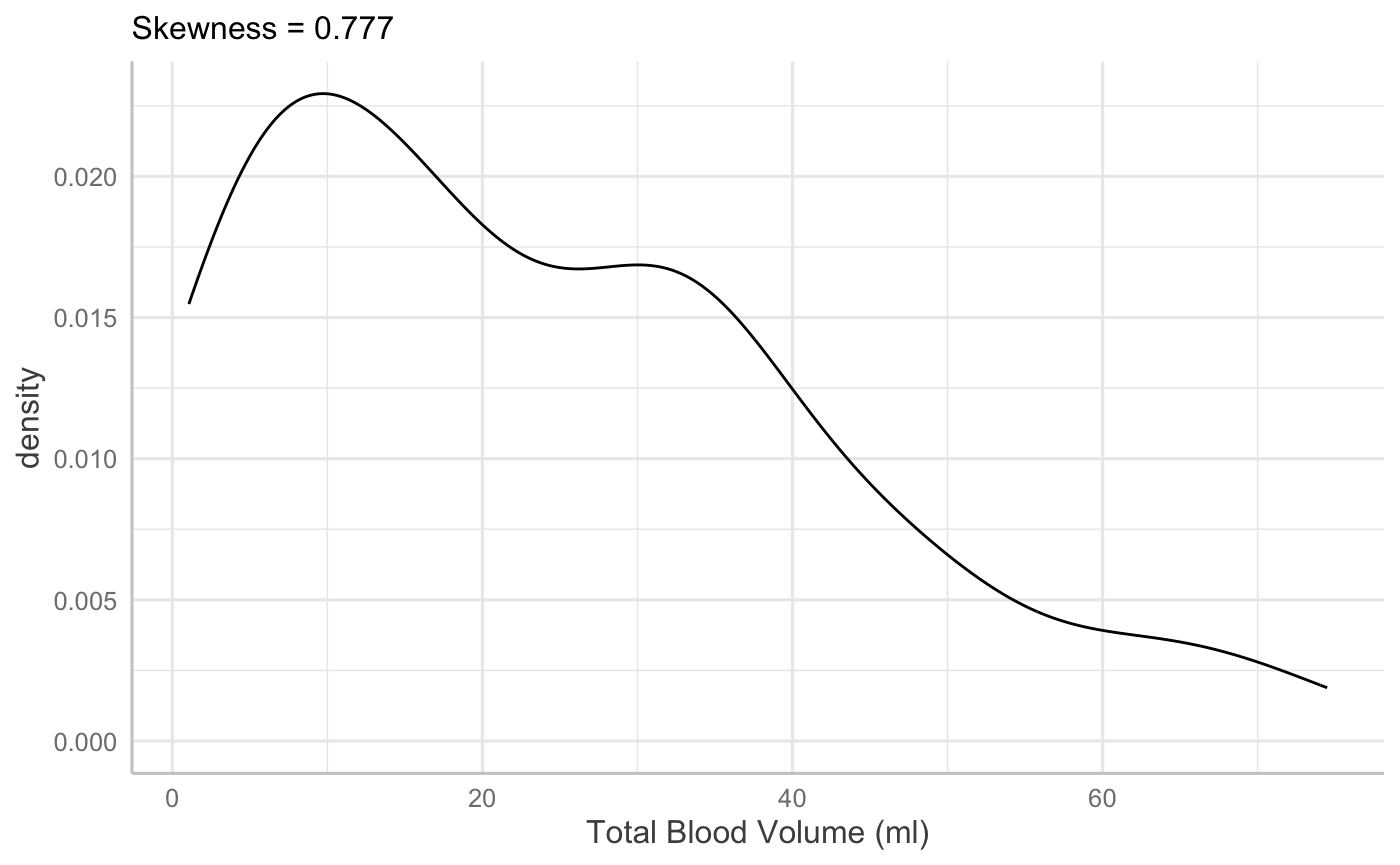


**Fig. 1. Distribution of Total Blood Volume.**


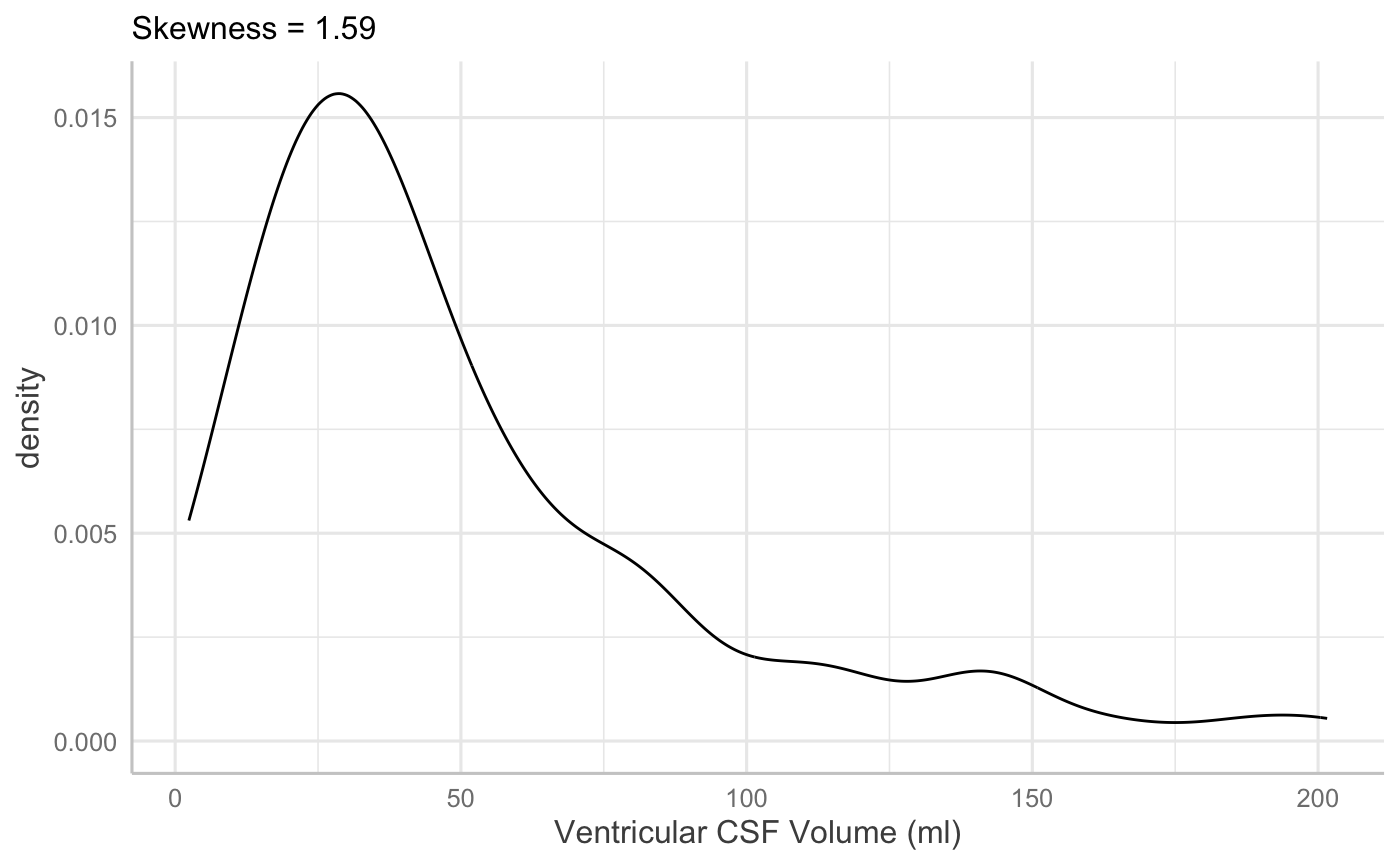


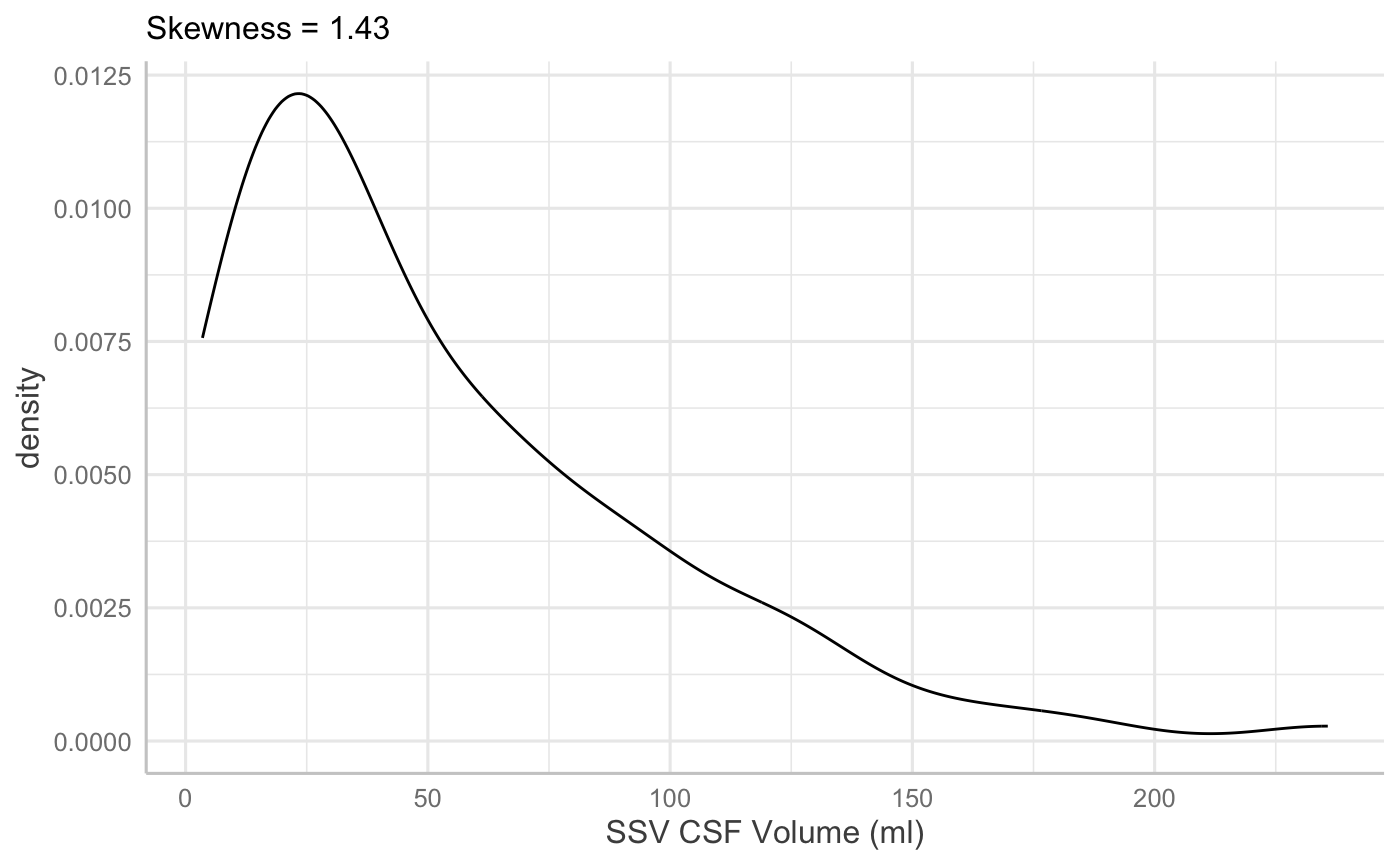


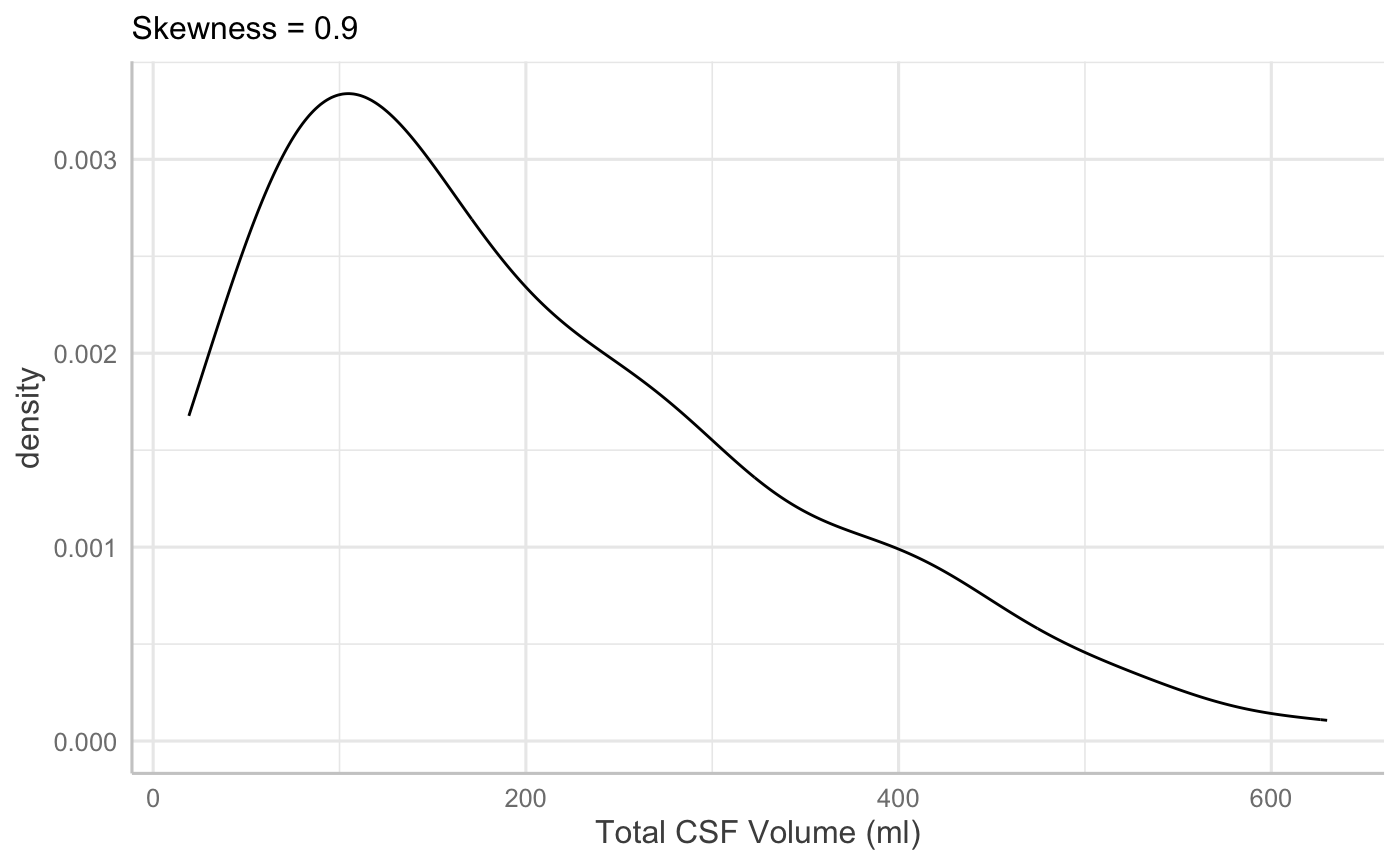


**Fig. 2. Distribution of CSF volume imaging variables.**
